# Oral Psoriasis of the Tongue: A Case Report

**DOI:** 10.7759/cureus.6318

**Published:** 2019-12-07

**Authors:** William J Ferris, Suzette Mikula, Ronald Brown, Andre Farquharson

**Affiliations:** 1 Orthopaedics, Georgetown University Hospital, Washington, D.C, USA; 2 Otolaryngology, Georgetown University Hospital, Washington, D.C, USA; 3 Dentistry, Howard University, Washington, D.C, USA; 4 Pathology, Howard University, Washington, D.C, USA

**Keywords:** psoriasis, tongue

## Abstract

Psoriasis is a common dermatological disease, but oral psoriasis is rarely reported in the literature. Its diagnosis has been a contentious issue among physicians. Its presence in the absence of skin lesions is not completely accepted by all physicians, and diagnosis is complicated by the fact that there are no defined criteria. We present a case report of oral psoriasis in a man who did not have skin lesions concurrently but did have a previous history of dermal psoriasis. Additionally, we discuss the history, typical presentation, and current treatments of oral psoriasis.

## Introduction

Psoriasis is a relatively common immune-mediated, chronic, genetically determined, scaly inflammatory disease that primarily affects the skin and secondarily the joints. Various sources have noted that the prevalence rate of this condition ranges between 0.5-4.6% of the population worldwide. The most common form of psoriasis is the plaque type, also known as psoriasis vulgaris, which accounts for approximately 90% of occurrences. The most common anatomical presentations of these lesions occur on the elbows, knees, scalp, fingernails, and toenails [1-5].

Oral psoriasis of the tongue is an extremely rare condition. Oppenheim first reported on the histopathology of oral psoriasis in 1903 [6]. In 1997, Younai and Phelan reported that only 57 cases met the criteria to be confirmed as cases of oral psoriasis. Lier et al. (2009) reported that the occurrence of true psoriatic oral mucosal lesions had been disputed in the past and only seven more cases had been identified as of 2009. Of these 64 reported cases, only 11 have demonstrated characteristic psoriatic lesions of the tongue, and in five of these 11 cases, cutaneous psoriatic lesions were not present [7]. Mattsson et al. (2015) noted that the diagnosis of the oral mucosal psoriatic lesion is problematic as there are no clinical or histopathologic criteria. Another factor that further complicates the diagnosis is the variable appearance of lesions diagnosed as oral mucosal psoriatic lesions [4].

Van der Wall and Pindborg (1986) described four types of clinical presentations of oral psoriasis: 1) well-defined, gray to yellowish-white, very small roundish lesions; 2) lacy, circinate, elevated white lesions of the oral mucosa, including the tongue, which paralleled cutaneous lesions; 3) fiery-red erythematous oral lesions corresponding with acute cutaneous lesions; and 4) benign migratory glossitis (BMG), which occurs more frequently in patients with cutaneous psoriasis [8].

The association of psoriasis with both fissured tongue and BMG (also known as geographic tongue) is well documented. The prevalence of fissured tongue ranges between 9.8-47%, and the prevalence of BMG ranges between 5.6-8.1% in patients with cutaneous psoriasis [2,9]. The prevalence of BMG overall is between 1-2% of the population [10]. Picciani et al. reported that the oral condition called fissured tongue has an incidence of between 5-10% among the global population [9].

## Case presentation

**Figure 1:**
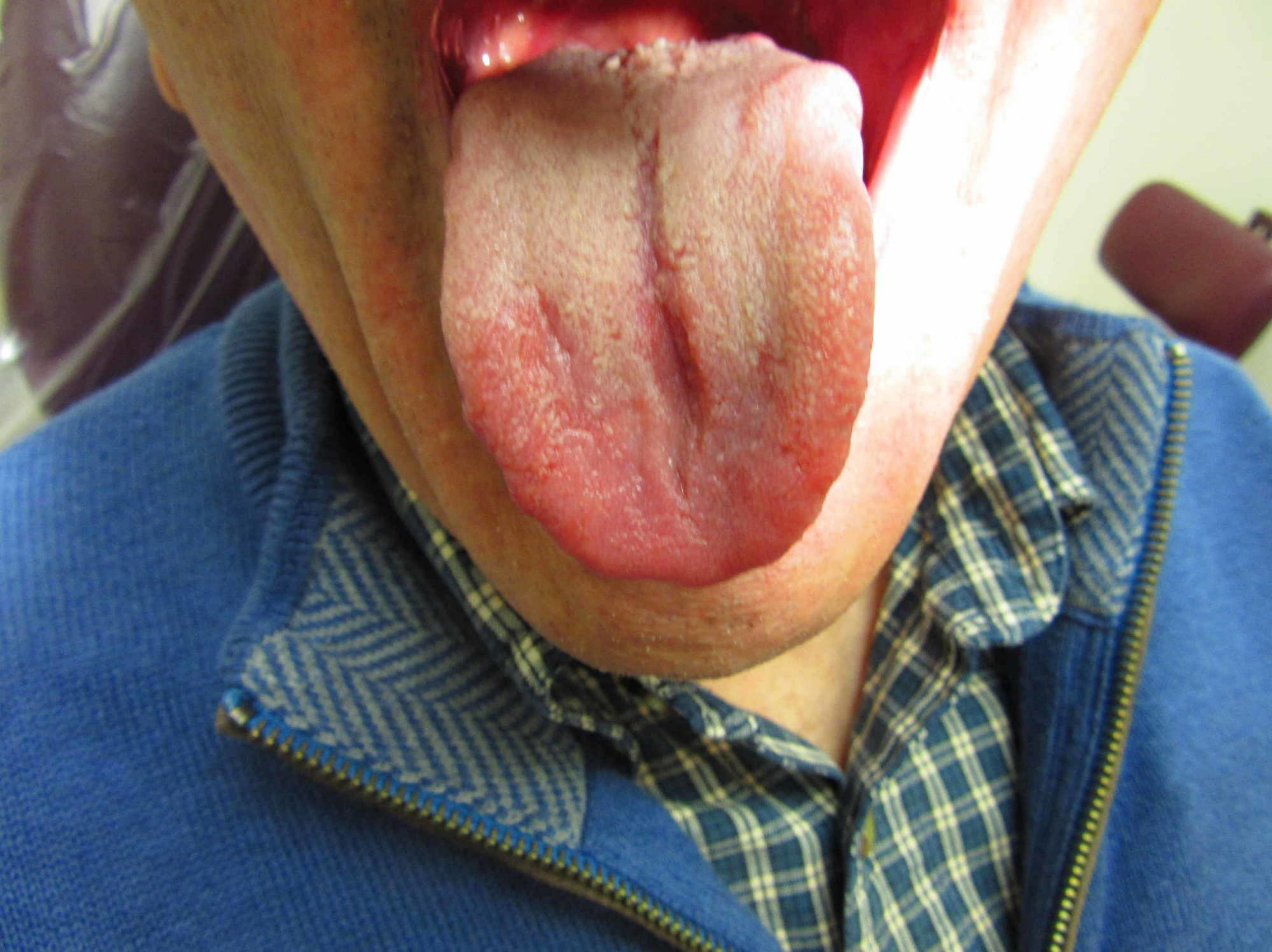
The condition of the patient's tongue before treatment

A 66-year-old patient presented to a private oral medicine clinician in early November 2017 with a chief complaint of “burning tongue, red at the tip, white bumps in the rear, cuts in tongue lesion, and bad taste.” His physician had previously placed him on a nystatin topical rinse for one week without any resolution of the condition. The condition had been ongoing for approximately two and a half months and the patient also reported difficulty in speaking. The patient had replaced his usual toothpaste with a toothpaste that was supposedly free of sodium lauryl sulfate. Eating spicy foods was not problematic. The patient’s pain was negligible upon waking up but increased as the day progressed. The condition did not interfere with his sleep. The patient was taking apixaban for atrial fibrillation, atorvastatin for cholesterol, lansoprazole for gastric reflux, and fexofenadine and azelastine for seasonal allergies, along with a vitamin D supplement. The patient reported no known drug allergies. Clinically, no lymphadenopathy was noted. The anterior dorsal tongue was noted for erythema, and the remaining oral tissues appeared to be within normal limits (Figure 1). 

**Figure 2:**
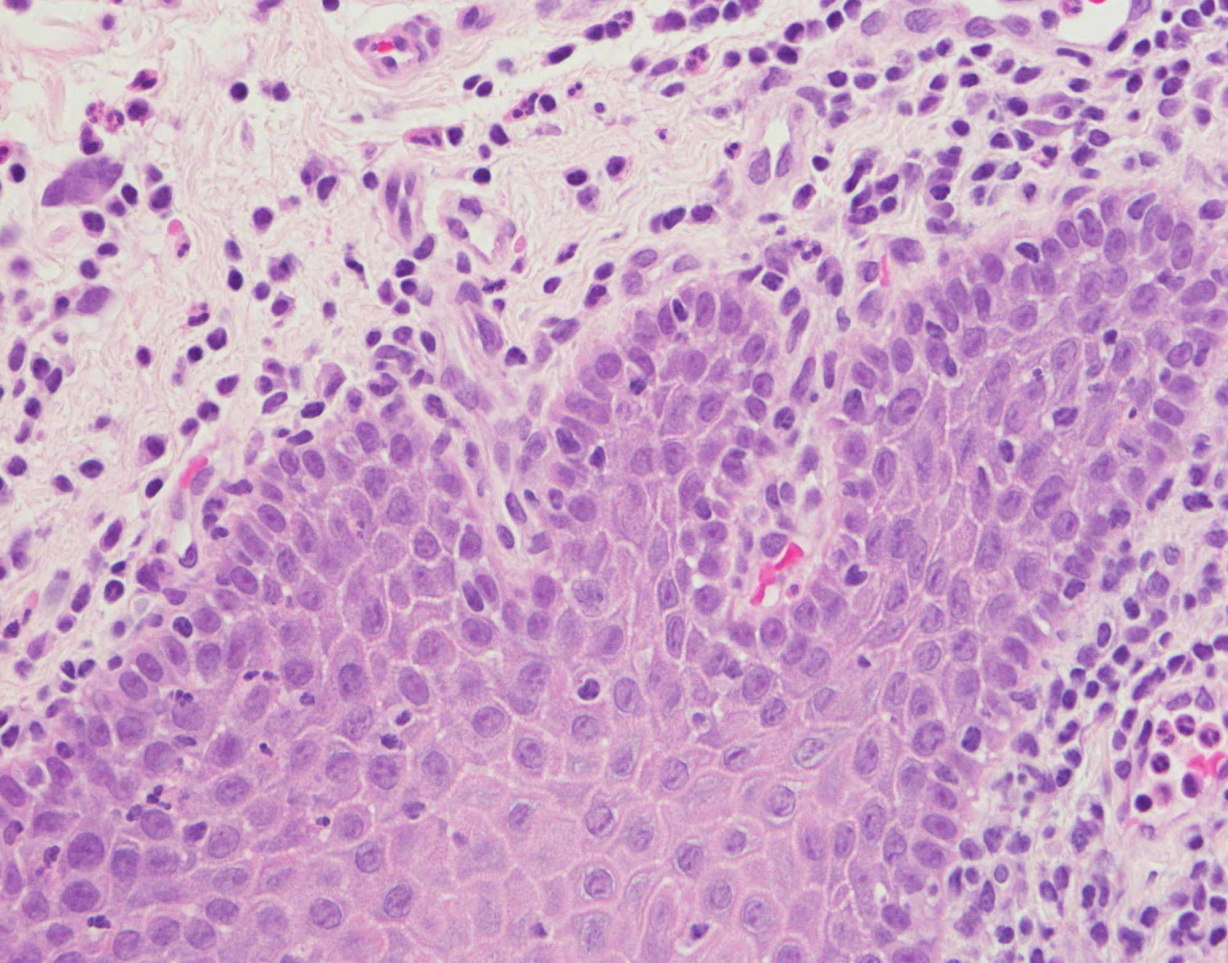
Histopathology of the tongue with elongated rete pegs

**Figure 3:**
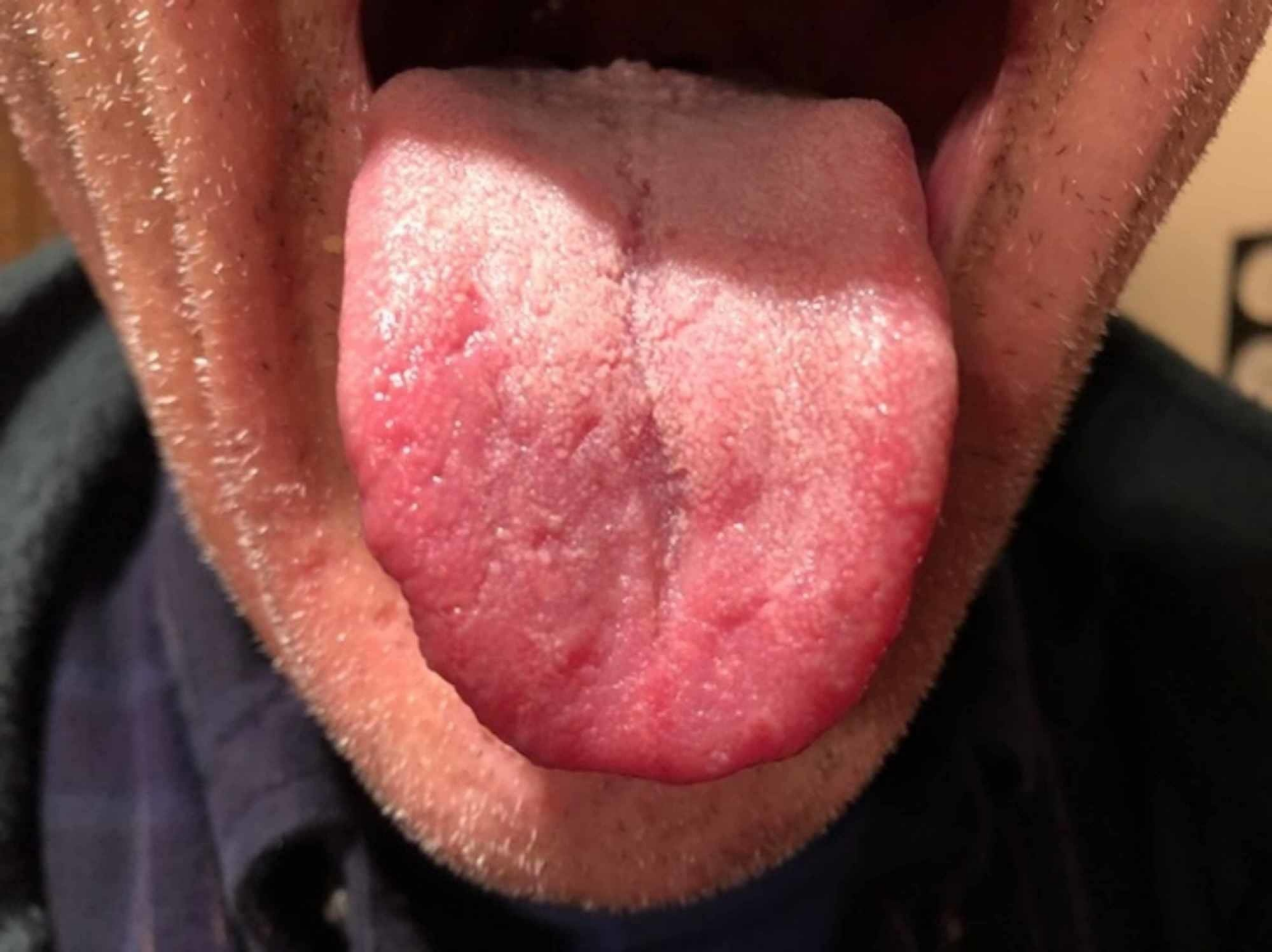
Worsening condition after treatment with antifungal

**Figure 4:**
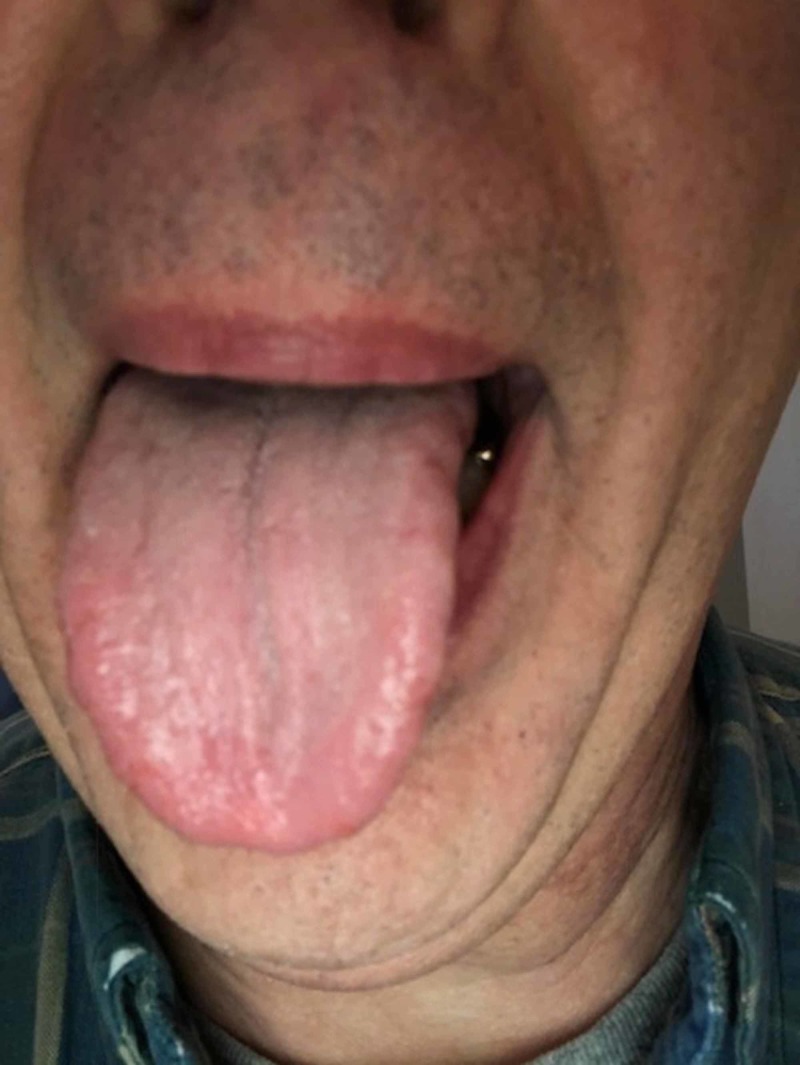
Condition resolved with steroid treatment

**Figure 5:**
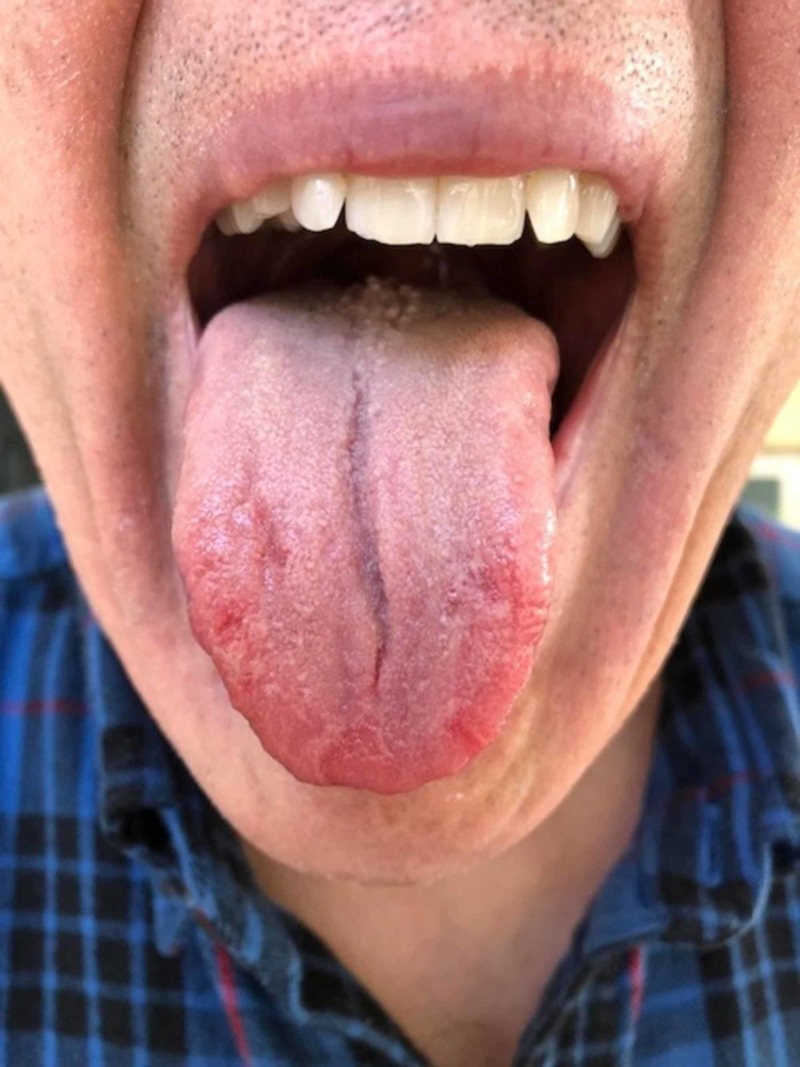
Condition returned after discontinuing steroid

The differential diagnosis consisted of hypersensitivity reaction, irritation reaction, oral candidiasis, and dysgeusia. The patient was referred to an allergy and immunology physician for food hypersensitivity evaluation in late November, and the evaluation studies were negative. The patient reported that his tongue seemed to be doing better and he suspected the reason was that he had removed paprika from his diet. However, the patient’s oral burning symptoms returned in January and he was scheduled for a biopsy procedure. The biopsy procedure and histological examination were performed in late January 2018. The pathology was positive for periodic acid-Schiff (PAS) staining for candidiasis. The histopathology slide at medium magnification exhibited elongated rete pegs (Figure 2). The papillary connective tissue was noted for lymphocytic inflammation and dilated blood vessels approximating the epithelial margins. Lower magnification showed marked collections of neutrophils seen in the parakeratin, which was consistent with Munro abscesses. The patient was placed on a two-week regimen of 100 mg fluconazole tablets daily. But the patient's condition further deteriorated (Figure 3). The patient was then referred to otolaryngology for a fungal DNA identification culture and sensitivity assay. The culture was negative for candida, aerobic, anaerobic, or acid-fast bacilli. The otolaryngologist elucidated that the patient had previously been treated for cutaneous psoriasis, and the patient also reported a history positive for psoriasis which had begun in his teens. The patient reported that he had had dry red blotchy lesions of the legs, trunk, and face. His dermatologist had prescribed calcipotriene ointment for the trunk and leg lesions and desonide for his facial lesions. The medications had been sufficient to control the condition. The patient did not remember having psoriatic lesions when the tongue lesions first presented in the late summer of 2017, although he was not sure whether there had been cutaneous psoriatic lesions at that time. The oral medicine clinician came up with a new working diagnosis of oral psoriasis and prescribed a regimen of topical dexamethasone elixir rinse. The patient’s lesions subsequently resolved (Figure 4). The patient was asked to discontinue the steroid rinse as a challenge and, in two weeks, the erythema and sensitivity returned (Figure 5). On resuming the topical steroid regimen, the lesions once again resolved. The patient was referred to his dermatologist to consider a biologic therapeutic, but the dermatologist deferred as he felt that the topical rinse seemed to be effective and was a more conservative approach.

## Discussion

As demonstrated by this case, oral psoriasis lesions are often misdiagnosed for other, more common pathologies. A definitive diagnosis can be challenging due to several factors including an unclear etiology, ill-defined clinical and histopathologic criteria, and rare occurrence with a variety of presentations. A differential diagnosis for any suspected oral psoriasis lesion should include lichen planus, syphilis, lupus erythematous, cicatricial pemphigoid, pemphigus, candidiasis, reactive arthritis, and smoking or trauma [11]. The correct diagnosis, in this case, relied on a careful study of the patient's history, physical exam, and biopsy with histological examination.

Most oral psoriasis lesions appear in the context of cutaneous lesions, either presenting simultaneously or appearing in patients with a history of cutaneous psoriasis [12]. A careful analysis of the patient's history is crucial to elucidating such crucial details. After the patient's history is examined, it is imperative to conduct a thorough exam of the patient’s skin for psoriatic lesions. Such lesions are often asymptomatic and may be in progression or regression unbeknownst to the patient. Identification of any cutaneous psoriatic lesions would elevate the diagnosis of oral psoriasis in the differential.

Isolated cases of oral psoriasis have been reported and the diagnosis cannot be ruled out based on a negative history and physical exam alone. A biopsy with periodic acid-Schiff-diastase (PAS-D) staining is often useful to distinguish between a superficial fungal infection and psoriasis. In general, pathologic changes seen within the mucous membranes parallel those of cutaneous psoriasis-elongation and thickening of rete ridges with overall acanthosis. The papilla of the lamina propria is elongated and edematous [11]. More specific immunohistochemical staining for factors such as vascular endothelial growth factor (VEGF) and tumor necrosis factor (TNF) is useful in a more definitive diagnosis of oral psoriatic lesions [13]. Even so, as reported in this case, initial biopsies may be misleading and continued follow-up and flexibility in treatment are fundamental to arriving at the correct diagnosis. In the case presented, the biopsy initially reported inflamed oral mucosa with candidiasis. However, after refractory antifungal treatment, a subsequent biopsy was performed that was negative for candidiasis, which led the clinician to inquire more about possible cutaneous psoriasis whereupon the patient revealed a history of dermal psoriasis in his teenage years. Patients with dermal psoriasis are known to be more susceptible to oral psoriasis or geographic tongue. Histologically, oral psoriasis and geographic tongue are similar in characteristics but occur in different locations. Often, geographic tongue exhibits a prominent white serpentine border and erythematous center while oral psoriasis has a white border that is either less prominent or not present at all.

Management of oral psoriasis covers a spectrum of treatments ranging from nonintervention to biomarker testing and biologic therapy to skin grafting. For asymptomatic lesions, treatment is not necessary and, undoubtedly, many of these lesions are unreported. Commonly, oral psoriasis can cause erythema, bleeding, plaque, or ulcers, and symptoms of discomfort such as pain, loss of taste, and hypersensitivity. For irritant-driven lesions, lifestyle modifications that focus on removing the irritant are preferred. Common irritants include spicy foods, smoking, and abrasive dentures or teeth. For nonirritant-related lesions, first-line treatment typically centers around palliative care using topical anesthetics such as viscous lidocaine, diphenhydramine, and alkaline rinses, which have all been reported to provide relief. Corticosteroids are also useful in reducing inflammation and suppressing the migration of polymorphonuclear lymphocytes [12]. Often, regression of the psoriatic lesion is observed and such patients have a good prognosis in the long term. For patients refractory to this care, advances in the understanding of cytokines and inflammatory disease processes have introduced a new field in the therapeutic treatment of psoriasis. Historically, anti-TNF agents developed for rheumatology and gastroenterology have been used with some success in treating psoriasis. Etanercept, introduced in 2004, was the first Food and Drug Administration (FDA)-approved biologic for dermatologic treatment and has a safe and effective record [14]. Recently, significant progress has been made in understanding the role of the Interleukin-23 (IL-23)/T-helper 17 (Th17) signaling pathway in immune-mediated diseases. Biologics such as ixekizumab and secukinumab that disrupt Interleukin-17 (IL-17), or its receptor in the case of brodalumab, are highly effective and safe in treating moderate to severe psoriasis [14]. To conclude, these therapies are primarily intended to treat dermal psoriasis, but with the close relation and often co-presentation of both dermal and oral psoriasis, the clinician may consider their use in severe oral psoriasis. In the case of our patient, biologic treatment was not pursued since the topical rinses were effective.

Currently, research is being conducted in the field of biomarkers for the strategic diagnosis and treatment of psoriasis. Unfortunately, no biomarkers specific to psoriasis of any type have been identified so far. However, researchers remain hopeful that biomarkers will yield novel strategies and treatments to improve patient management and outcomes [5]. Surgical procedures such as gingival grafting should be reserved for candidates refractive to medical therapy.

Lastly, consideration must be given to the simultaneous presentation of oral psoriasis with another disease on the differential, most often candidiasis. While the treatment of many oral lesions is straightforward, an astute clinician must always consider the potential overlap of multiple disease processes, which may complicate both the diagnosis and treatment.

## Conclusions

While rarely reported, oral psoriasis is a diagnosable lesion with effective and adequate treatments. The patient presented follows the classic storyline of misdiagnoses and mistreatment that often happens due to a misleading biopsy and/or incomplete history. Although cases of isolated oral psoriasis have been reported, patient history or physical-exam findings pertaining to cutaneous psoriasis is a notable clue in diagnosing oral psoriasis. Treating oral psoriasis should be pursued in a gradual, step-wise fashion, beginning with irritant removal and gradually advancing to palliative care and eventually biologic therapy based on the requirement. Grafting and other surgical procedures should be considered after all the medical treatment avenues have been considered. To sum up, oral psoriasis is an uncommon ailment that can be effectively diagnosed and treated through common medical practices including a thorough study of the patient history, methodical physical exam, confirmatory testing, and continued patient-physician dialogue.
